# Construct validation of the Health Utilities Index and the Child Health Questionnaire in children undergoing cancer chemotherapy

**DOI:** 10.1038/sj.bjc.6600895

**Published:** 2003-04-15

**Authors:** L Sung, M L Greenberg, J J Doyle, N L Young, S Ingber, J Rubenstein, J Wong, T Samanta, M McLimont, B M Feldman

**Affiliations:** 1 Department of Health Policy Management and Evaluation, University of Toronto, Toronto, Ontario, Canada; 2 Division of Hematology/Oncology, Hospital for Sick Children, Toronto, Ontario, Canada, M5G 1X8; 3 Department of Pediatrics, Population Health Sciences Program, Hospital for Sick Children, Toronto, Ontario, Canada, M5G 1X8; 4 Division of Rheumatology, Hospital of Sick Children, Toronto, Ontario, Canada, M5G 1X8; 5 Department of Public Health Science, University of Toronto, Toronto, Ontario, Canada

**Keywords:** chemotherapy, pediatric, health-related quality of life, Health Utilities Index, Child Health Questionnaire

## Abstract

The objective of this study was to evaluate the construct validity of two questionnaire-based measures of health-related quality of life (HRQL) in children undergoing cancer chemotherapy: the Health Utilities Index (HUI) and the Child Health Questionnaire (CHQ). Subjects were children hospitalised for chemotherapy. To examine construct validity: (1) *a priori* expected relations between CHQ concepts and HUI attributes were examined; (2) HUI and CHQ summary scores were compared to visual analogue scale (VAS) scores. Ease of completion was rated using a 5-point categorical scale and completion time was recorded. A total of 36 subjects were included. The maximum score was seen in 15 (47%) of HUI3 assessments. As predicted, CHQ body pain was moderately correlated with HUI3 pain (*r*=0.51), CHQ physical functioning was moderately correlated with HUI2 mobility (*r*=0.58) and CHQ mental health was moderately correlated with HUI2 emotion (*r*=0.53). Only the CHQ psychosocial subscale (and not HUI) was correlated with VAS (*r*=0.44). The CHQ and the HUI were both easy to use. The HUI questionnaires required less time to complete (mean=3.1, s.d.=1 min) compared with CHQ (mean=13.1, s.d.=3.4 min, *P*<0.0001). In conclusion, HUI and CHQ demonstrated construct validity in children undergoing cancer chemotherapy. The Health Utilities Index is subject to a ceiling effect whereas CHQ requires more time to complete.

The health-related quality of life (HRQL) of children undergoing chemotherapy for cancer is becoming increasingly emphasised in clinical trials. Understanding differences in HRQL associated with different treatment strategies may be particularly helpful to families and health-care workers when these strategies are associated with similar survival.

Some suggest that both generic and disease-specific instruments should be used in the assessment of HRQL (Guyatt *et al*, 1993). The advantage of generic measures is that they provide a rating of quality of life that permits comparisons across illnesses and often have normative reference data (Spieth and Harris, 1996). However, they may lack validity or responsiveness in specialised clinical subgroups such as children undergoing chemotherapy for cancer. We have been unable to find a comparison of generic instruments in this specific group.

We evaluated two questionnaire-based measures of HRQL, the Health Utilities Index (HUI) and the Child Health Questionnaire (CHQ). The rationale for choosing these measures is that both the HUI and CHQ have been used to evaluate survivors of childhood cancer (Feeny *et al*, 1992,^1993^; Billson and Walker, 1994; Kiltie and Gattamaneni, 1995; Glaser *et al*, 1997,1999a,1999b; Barr *et al* 1999; Sawyer *et al*, 1999; Speechley *et al*, 1999; Felder-Puig *et al*, 2000; Sands *et al*, 2001) and they were both incorporated into a cross-Canada study of the long-term psychosocial and physical health of childhood cancer survivors (The Late Effects Study) (Gibbons *et al*, 1994). Additionally, the HUI has been included in every major Canadian population health survey since, 1990 (Furlong *et al*, 2001). These questionnaires therefore allow comparison of HRQL between children receiving cancer chemotherapy and long-term cancer survivors as well as enabling comparisons to population estimates of health.

Although HUI and CHQ both measure HRQL, they have important differences. They are based on different theoretical approaches with the HUI being a utility-based measure of overall HRQL, while the CHQ is a health-profile measure using summative categorical scaling to determine separate scores in two subscales. Also, different frameworks are used. The HUI uses a narrow ‘within the skin’ approach to the measurement of HRQL and does not include social interactions (which are considered to reflect phenomenon other than the strict health of the individual) (Feeny *et al*, 1996). Conversely, the CHQ is broader in scope and emphasises some ‘outside the skin’ attributes such as behaviour and the impact of the child's health on behaviour.

There are three studies supporting the validity of the HUI in children receiving cancer chemotherapy. (Feeny *et al*, 1992; Barr *et al*, 1997; Trudel *et al*, 1998). These showed that the HUI was able to discriminate between children on and off treatment for cancer and was responsive to different phases of treatment in children receiving maintenance therapy for acute lymphoblastic leukaemia (ALL). The CHQ has not been studied in this population and consequently, this is the first study that concurrently examines both measures in children receiving chemotherapy for cancer.

Our primary objective was to examine the construct validity of the HUI and CHQ by (1) anticipating that domains within each instrument that are measuring similar concepts should be moderately correlated and (2) predicting that the HUI and the CHQ summary scores should show fair correlation with a global HRQL visual analogue scale (VAS). Our second objective was to examine the feasibility and ease of administration of these instruments.

## METHODS

### Subjects

This study was part of a larger study examining the measurement of HRQL in children with a variety of illnesses. The sample for the study reported here consisted of consecutive children aged between 1 and 18 years admitted to the Hospital for Sick Children for cancer chemotherapy. We did not want the acute effects of chemotherapy to influence the assessment of HRQL strongly; therefore, we collected data prior to the initiation of chemotherapy whenever possible and only included those subjects for whom data could be collected within 24 h of chemotherapy initiation. We excluded children admitted for their first cycle of chemotherapy, those receiving palliative chemotherapy and those where the respondent was non-English speaking.

### Study design

This study was approved by the Research Ethics Board at the Hospital for Sick Children and written consent was obtained from all participating families; children aged between 7 and 15 years gave verbal assent.

All study questionnaires were administered in an interview format by one of four trained research assistants. To ensure consistency in interviewing styles, these four initially piloted the questionnaires on each other and then working in pairs, performed 12 pilot interviews in which one research assistant administered the questionnaires and the other observed. Standardised scripts were used.

Parent report was used for all measurements. The parents were presented with the different HRQL measures in random order to control for an order effect at the group level of analysis. The HUI and VAS both used a 1-week recall period (the period of time that parents were asked to consider when rating different aspects of health), while the CHQ was only available with a 4-week recall period.

### Instruments

#### Health Utilities Index

The HUI is a family of multiattribute health status classification systems which currently consists of two complementary systems: HUI Mark 2 (HUI2) and HUI Mark 3 (HUI3). (Furlong *et al*, 2001). The HUI2 was developed to assess the health status of childhood cancer survivors (Feeny *et al*, 1992) and is composed of seven attributes: sensation, mobility, emotion, cognition, self-care, pain and fertility. Health Utilities Index Mark 3 was first used in the 1990 Ontario Population Health Survey and it is composed of eight attributes: vision, hearing, speech, ambulation, dexterity, emotion, cognition and pain. HUI2 describes 24 000 unique health states, while HUI3 describes 972 000 unique health states.

One of the attractive features of the HUI is that the health states defined by a comprehensive set of HUI levels can be used to determine single-attribute and overall utility scores. Utility can be defined as the strength of an individual's preference for a health state measured under conditions of uncertainty and is expressed on a continuous scale from 0 to 1 in which 0 represents death and 1 represents perfect health (Von Neumann and Morgenstern, 1953). Utility may be the best measure of HRQL for the purpose of decision or economic analyses since this measure incorporates the uncertainty and risk reflected in actual decision-making (Torrance *et al*, 2001). The functions for determining HUI utility scores have been published, and are based on preference measurements from adults in Hamilton, Ontario. (Torrance *et al*, 1996; Feeny *et al*, 2002).

This study used a standard 41-item English-language HUI® questionnaire for interviewer administration and proxy respondents (HUI23PIE.40Q). ‘Don't know’ and ‘refused’ responses were coded as missing, thus resulting in fewer respondents for whom there were complete data for HUI compared with other instruments.

#### Child Health Questionnaire

The CHQ is a multidimensional pediatric-specific HRQL comprehensive measure that encompasses 14 health concepts (Landgraf *et al*, 1996). These health concepts include physical functioning, bodily pain, general behaviour, mental health, self-esteem, general health perception and change in health and family cohesion. Other concepts are the impact of the illness on the emotional, behavioural and physical health of the child and the impact of the child's illness on the emotional health of the parent, on the activities of the family and on the time the parent has for personal issues.

The questionnaire responses can be used to generate two summary scores, the physical health (CHQ PhS) and psychosocial health (CHQ PsS) subscales. These scores are transformed such that in a general US population, they have a mean of 50 and a standard deviation of 10. We used the self-administered 50-item parent report version (PF-50).

#### Visual Analogue Scale

Respondents were asked to mark the HRQL of their child on a horizontal 10 cm line anchored at one end by death (score of 0) and the other end by perfect health (score of 1). There were no other increments marked on the line. Perfect health was described as meaning happiness and the ability to do the things one likes without pain or illness.

### Determination of construct validity

A valid instrument measures what it is purported to measure. In general, there are two ways that validity is assessed: criterion validity, in which the measure is compared against a ‘gold standard’ and construct validity, in which expectations about how a measure should behave are hypothesised and tested (Guyatt, 1999). Owing to the absence of a gold standard in the measurement of HRQL, these types of measures are usually assessed by construct validity.

One of the early steps in construct validation is to hypothesise how different measures should relate (convergent construct validity). The more an instrument behaves according to *a priori* hypothesised relations, the stronger is the evidence for validity (Guyatt, 1999).

We examined construct validity in two ways. First, we assessed *a priori* expected relation between CHQ health concepts and HUI single-attribute utilities according to Speechley *et al* (1999) who hypothesised the following in a group of childhood cancer survivors: (a) correlations between CHQ pain and HUI2 and HUI3 pain should be >0.50, (b) correlations between CHQ physical functioning and HUI2 mobility and HUI3 ambulation should be 0.35 – 0.50, (c) correlations between CHQ mental health and HUI2 and HUI3 emotion should be 0.35 – 0.50 and (d) correlations between CHQ general health perception and HUI2 and HUI3 global utilities should be 0.20 – 0.34. In that study of childhood cancer survivors, Speechley *et al* (1999) demonstrated relation that were similar or stronger than those hypothesised. A second study found similar relations in a predominantly healthy group of school children. (Raat *et al*, 2002).

The second way in which construct validity was assessed was by anticipating that HUI overall utility scores and CHQ summary scores should show fair correlation with the VAS described above. The correlations between scores should be positive since better health should score higher on all instruments. However, these correlations should not be strong because the weighting of different attributes of HRQL is predefined in the HUI and CHQ, whereas this weighting is subjectively performed by each respondent in the VAS.

### Assessment of ease of use

Respondents were asked to rate ease of use of each questionnaire on a 5-point categorical scale ranging from very easy to very hard. The start and stop times for completing each of the instruments were also recorded.

### Analysis

The scores were summarised by instrument using median value and interquartile range (IQR) because the scores were not normally distributed. Spearman correlations were used to evaluate the association between measures. Correlation coefficients were defined as follows: 0 – 0.25, negligible or not correlated; 0.25 – 0.50, fair correlation; 0.50 – 0.75, moderate-to-good correlation and >0.75, very good-to-excellent correlation (Colton, 1974).

The difference in the mean times to complete the HUI and CHQ was assessed using the paired Student's *t*-test. Statistical significance was defined as *P*<0.05 and multiple testing was addressed using Bonferroni adjustment where appropriate. All analyses were performed using the SAS statistical program (SAS-PC, Version 8.0; SAS Institute Inc., Cary, NC, USA).

## RESULTS

During the period from 1 June 2001 to 1 September 2001, we identified 42 eligible children. The parents of five refused and one parent did not complete the study because her child was interrupted by a diagnostic procedure. In total, 36 parents completed all the questionnaires. The mean parent respondent age was 37.7 (s.d.=5.6) years, while the mean age of their affected children was 7.2 (s.d.=4.0) years. Table 1

**Table 1:** Cancer diagnoses

**Diagnoses**	**Frequency (percent)**
Acute lymphoblastic leukaemia	12 (33.3)
Ewing's sarcoma	4 (11.1)
Germ cell tumour	3 (8.3)
Wilms tumour	1 (2.8)
Brain tumour	2 (5.6)
Lymphoma	2 (5.6)
Neuroblastoma	6 (16.7)
Osteosarcoma	1 (2.8)
Rhabdomyosarcoma	4 (11.1)
Adrenocortical carcinoma	1 (2.8)

shows the frequency of the diagnoses for these children: ALL was the most common diagnosis occurring in 12 (33%) of the children. The median time from the diagnosis of cancer to the interview date was 122.5 days (IQR 72.0, 247.5 days).

The median number of days between administration of the most recent cycle and the current cycle of chemotherapy was 28 days (IQR 22.5, 32 days). For the current chemotherapy admission, the number of hours of chemotherapy received prior to questionnaire administration was recorded; if chemotherapy had not yet begun at the administration of the questionnaire, this value was recorded as 0. The median time on chemotherapy at questionnaire commencement was 12.4 h (IQR 0.1, 20.8 h).

Table 2

**Table 2:** Distribution of summary scores

		**Maximum**	**Minimum**
**Measure (*n*)**	**Median (IQR)**	**No. (%)**	**No. (%)**
*Summary scores*			
CHQ PsS (36)	46 (39, 52)	0	0
CHQ PhS (36)	34 (21, 45)	0	0
HUI2 (32)	0.93 (0.80, 1.00)	11 (34)	0
HUI3 (32)	0.93 (0.76, 1.00)	15 (47)	0
VAS (35)	0.76 (0.58, 0.84)	0	0
			
*CHQ Concepts*			
Single-item general health (36)	60 (30,85)	5 (14)	4 (11)
General health perception (36)	52 (43, 66)	0	0
Physical functioning (36)	75 (50, 92)	7 (19)	0
Bodily pain (36)	60 (40, 80)	5 (14)	0
Single-item global behaviour (36)	85 (60,100)	11 (31)	1 (3)
General behavior (36)	77 (65, 86)	2 (6)	0
Mental health (36)	73 (65, 80)	1 (3)	0
Self-esteem (36)	75 (56, 85)	2 (6)	0
Family activities (36)	50 (25, 67)	0	0
Family cohesion (36)	85 (85, 85)	6 (17)	0
Parent time impact (36)	56 (33, 67)	2 (6)	3 (8)
Parent impact emotional (36)	42 (25, 50)	1 (3)	5 (14)
Role–physical (36)	67 (33, 100)	10 (28)	5 (14)
Role–emotional/behaviour (36)	100 (67, 100)	20 (56)	0
			
*HUI2 single-attribute utilities*			
Sensation (34)	1 (1, 1)	28 (82)	0
Mobility (35)	1 (1, 1)	29 (83)	0
Emotion (36)	1 (0.86, 1)	20 (56)	3 (8)
Cognition (34)	1 (0.86, 1)	25 (74)	0
Self-care (35)	1 (1, 1)	34 (97)	1 (3)
Pain (36)	1 (0.95, 1)	25 (69)	0
			
*HUI3 single-attribute utilities*			
Vision (35)	1 (1, 1)	33 (94)	0
Hearing (36)	1 (1, 1)	36 (100)	0
Speech (35)	1 (1, 1)	31 (89)	0
Ambulation (35)	1 (1, 1)	32 (91)	0
Dexterity (36)	1 (1, 1)	35 (97)	0
Emotion (36)	1 (1, 1)	31 (86)	0
Cognition (34)	1 (0.92, 1)	25 (74)	0
Pain (36)	1 (0.92, 1)	25 (69)	0

reports the median scores and the frequency of minimum and maximum scores for each instrument including the individual CHQ concepts and HUI single-attribute utilities. The maximum possible score (ceiling effect) was common with the HUI but was not seen with the CHQ summary scores.

Table 3

**Table 3:** Convergent validity of CHQ and HUI

**CHQ concept**	**HUI2 attribute and correlation coefficient observed**	**HUI3 attribute and correlation coefficient observed**	**Correlation coefficient predicted^a^**
Bodily pain	Pain, 0.50*(0.21, 0.71)	Pain, 0.51*(0.22, 0.72)	>0.50
Physical functioning	Mobility, 0.58*(0.31, 0.77)	Ambulation, 0.43 (0.12, 0.67)	0.35 – 0.50
Mental health	Emotion, 0.53*(0.24, 0.73)	Emotion, 0.37 (0.04, 0.62)	0.35 – 0.50
General health perceptions	Global HUI2, 0.29 (−0.06, 0.58)	Global HUI3, 0.36 (0.01, 0.63)	0.20 – 0.34

a Spearman correlation coefficients (95% confidence intervals). Criteria adapted from Speechley *et al* (1999).

* *P*-values < 0.006 (Bonferroni adjustment for multiple testing).

presents the correlations between selected subscale scores of CHQ and single-attribute utilities of HUI. As anticipated, CHQ body pain was moderately correlated with HUI3 pain (*r*=0.51; *P*=0.001), CHQ physical functioning was moderately correlated with HUI2 mobility (*r*=0.58; *P*=0.0003) and CHQ mental health was moderately correlated with HUI2 emotion (*r*=0.53; *P*=0.001). If adjustment for multiple testing had not been conducted, all correlation coefficients in Table 3 would have been statistically significant (*P*<0.05) except for the correlation between CHQ general health perception and the HUI2 summary score. In comparing HUI and CHQ summary measures, HUI2 and HUI3 overall multi-attribute scores were both moderately correlated with CHQ PhS (*r*=0.55, *P*=0.001 and *r*=0.50, *P*=0.004 respectively) but were not significantly correlated with PsS (*r*=0.32 and *r*=0.09, both *P*=NS).

Table 4

**Table 4:** Correlation coefficients between HUI and CHQ summary scores with VAS^a^

	**HUI2**	**HUI3**	**CHQ PhS**	**CHQ PsS**
VAS	0.04	0.01	0.32	0.44*
	(−0.32, 0.39)	(−0.34, 0.37)	(−0.02, 0.59)	(0.13, 0.68)

a Spearman correlation coefficients (95% confidence intervals).

**Figure 1:**
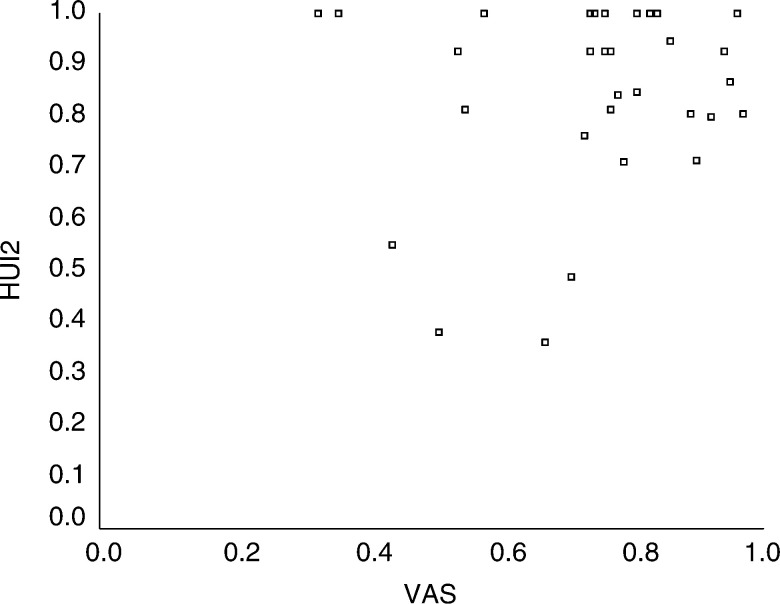
Scatterplot of HUI2 and VAS. The *y*-axis represents the scores on the HUI2 where 0 represents HRQL equal to death and 1 represents HRQL equal to perfect health. The *x*-axis represents the scores on the global HRQL VAS with the same anchors.

**Figure 2:**
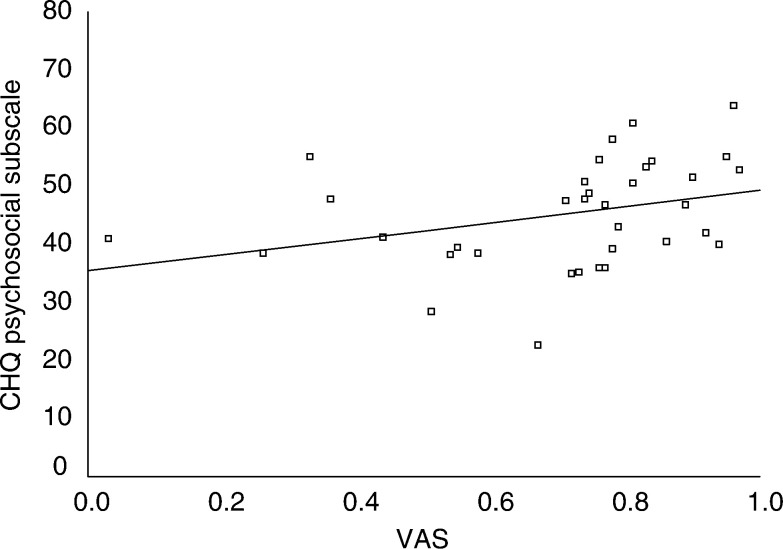
Scatterplot of CHQ PsS and VAS. The *y*-axis represents the scores on the CHQ PsS in which higher scores reflect better HRQL. The *x*-axis represents the scores on the global HRQL VAS in which 0 is equal to death and 1 is equal to perfect health. The line represents the least-squares regression line.

* *P*<0.01 (Bonferroni adjustment for multiple testing).

presents the relations between HUI and CHQ summary scores and the HRQL VAS. HUI2 and HUI3 were not correlated with VAS as illustrated in Figure 1

(for HUI2). We were unable to fit the data using nonlinear transformations. Conversely, PsS showed fair correlation with VAS (*r*=0.44, *P*=0.008); this relationship is demonstrated in Figure 2

.

In trying to determine whether the child's age influenced the above findings, construct validation was repeated for the 24 children who were 5 years of age or greater. The findings were unchanged.

Figure 3

**Figure 3:**
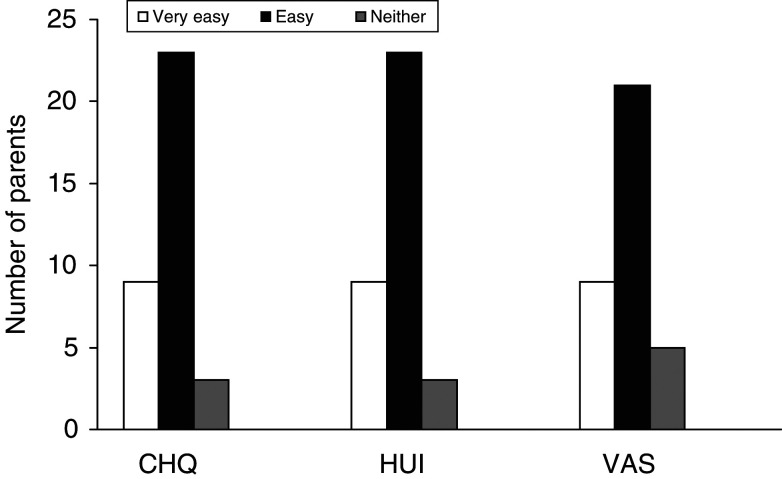
Bar graph illustrating the ease of completion of the different measures using a 5-point categorical scale. No-one rated any instrument as ‘difficult’ or ‘very difficult’ to complete.

illustrates the ease of completion for all instruments. The HUI and CHQ were easy to complete with 32 out of 36 (89%) of parents stating that the questionnaires were easy or very easy. Completion time was significantly shorter for the HUI (mean=3.1, s.d.=1 min) compared to the CHQ (mean=13.1, s.d.=3.4 min, *P*<0.0001).

## DISCUSSION

In this study, we have shown that CHQ and HUI demonstrate construct validity in children undergoing chemotherapy as the CHQ health concepts and HUI single-attribute utility scores were related according to *a priori* expectations. However, while the CHQ PsS subscale was significantly correlated with VAS, HUI was not correlated with VAS. There are at least two possible reasons why CHQ and HUI may have different relation with VAS. First, both CHQ and VAS measure phenomena ‘outside the skin’ and thus, may be measuring a more ‘global’ construct of health compared to HUI. Second, disparity in HUI and VAS may arise because HUI utilities reflect mean community preferences about a health state defined by a well-specified classification system. In contrast, VAS reflects a parent's opinion about their particular child's subjectively defined health state that may not be definable in terms of an HUI system.

We have also demonstrated that it is feasible to recruit parents as proxy respondents and obtain data on HRQL while children are receiving chemotherapy for cancer. Consequently, this study has important implications regarding the incorporation of HRQL measures in clinical trials within paediatric oncology. Parents are an important source of HRQL measurement in pediatric cancer trials as many of the affected children are too young for self-reporting. However, when parents are the respondents, it is possible that factors such as the emotional state and health of the parent can affect their assessment of their child's HRQL.

In deciding which measure should be incorporated into a clinical trial, several factors should be considered. The advantages of the HUI are that it is simple to use and requires little time to complete. The CHQ is similarly easy to use and is not subject to a ceiling effect, although it does require more time. The Health Utilities Index is preferable for the purposes of decision or economic analyses because of the expression of HRQL as a utility. The HUI may also be preferable if the purpose of the trial is to capture elements of HRQL that are restricted to ‘within the skin’ phenomena. However, if a more global measure of HRQL is preferred, then CHQ may be better.

The HUI may be problematic if incorporated into a trial attempting to improve the HRQL of children receiving an intensity of chemotherapy similar to our cohort because of its ceiling effect. We hypothesised that the HRQL of children receiving such chemotherapy is likely diminished between chemotherapy cycles related to common toxicities of treatment such as fatigue, fever and mucositis in addition to the emotional burden of having a life-threatening diagnosis; despite this many of the children had perfect HRQL according to HUI.

Our conclusions must be considered in the light of at least three limitations. First, although our study does represent the largest study evaluating either the HUI or CHQ in children receiving cancer chemotherapy, the sample size was small. While the correlation between CHQ physical functioning and HUI3 ambulation, and the correlation between CHQ mental health and HUI3 emotion were within the predicted ranges, the correlations were not statistically significant after adjustment for multiple testing, most likely related to insufficient statistical power. However, we did see a wide variety of different HRQL experiences in our study and were able to show several statistically significant correlations between the measures, suggesting that our sample size was adequate for our purposes.

The second limitation was the age of the children included in this study; 33% were less than 5 years old, reflecting the age distribution of children admitted for cancer chemotherapy. Both the HUI and CHQ are recommended for children of age greater than 5 years (Landgraf *et al*, 1996; Furlong *et al*, 2001). However, two of the studies examining the HUI in children undergoing anticancer therapy included children under the age of 5 years (Barr *et al*, 1997; Trudel *et al*, 1998); and specifically, the report by Barr *et al* (1997) included children as young as 11 months with a median age of 3 years 11 months. Both studies demonstrated that the HUI was reliable and valid in this population and age range. Furthermore, the robustness of our findings when the analysis was limited to those greater than 5 years of age further suggests that our results are valid even though young children were included. Nonetheless, our results must be cautiously interpreted in light of the age of our population.

Finally, the recall period for CHQ and HUI differed, as the CHQ was only available with a 4-week recall period. While we were reassured that the correlation between domains fell within the expected ranges, it is possible that there were real differences between the children's HRQL during the past week compared to the past 4 weeks. Validation of the CHQ with a 1-week recall period or validation of the HUI with a 4-week recall period in children undergoing cancer chemotherapy may be useful.

In summary, in children undergoing chemotherapy for cancer, HUI and CHQ demonstrate construct validity and either measure is appropriate to use in studies of HRQL. Both are easy to use although the HUI is subject to a ceiling effect while CHQ requires more time.
